# Nanoparticle Delivered Anti-miR-141-3p for Stroke Therapy

**DOI:** 10.3390/cells10051011

**Published:** 2021-04-25

**Authors:** Karishma Dhuri, Rutesh N. Vyas, Leslie Blumenfeld, Rajkumar Verma, Raman Bahal

**Affiliations:** 1 School of Pharmacy, University of Connecticut, Storrs, CT 06269, USA; karishma.dhuri@uconn.edu; 2 Department of Neurosciences, UConn Health, Farmington, CT 06032, USA; rvyas@uchc.edu (R.N.V.); lblumenfeld@uchc.edu (L.B.)

**Keywords:** ischemic stroke, peptide nucleic acid, phosphorothioates, anti-miR-141-3p, nanoparticles

## Abstract

Ischemic stroke and factors modifying ischemic stroke responses, such as social isolation, contribute to long-term disability worldwide. Several studies demonstrated that the aberrant levels of microRNAs contribute to ischemic stroke injury. In prior studies, we established that miR-141-3p increases after ischemic stroke and post-stroke isolation. Herein, we explored two different anti-miR oligonucleotides; peptide nucleic acid (PNAs) and phosphorothioates (PS) for ischemic stroke therapy. We used US FDA approved biocompatible poly (lactic-co-glycolic acid) (PLGA)-based nanoparticle formulations for delivery. The PNA and PS anti-miRs were encapsulated in PLGA nanoparticles by double emulsion solvent evaporation technique. All the formulated nanoparticles showed uniform morphology, size, distribution, and surface charge density. Nanoparticles also exhibited a controlled nucleic acid release profile for 48 h. Further, we performed in vivo studies in the mouse model of ischemic stroke. Ischemic stroke was induced by transient (60 min) occlusion of middle cerebral artery occlusion followed by a reperfusion for 48 or 72 h. We assessed the blood-brain barrier permeability of PLGA NPs containing fluorophore (TAMRA) anti-miR probe after systemic delivery. Confocal imaging shows uptake of fluorophore tagged anti-miR in the brain parenchyma. Next, we evaluated the therapeutic efficacy after systemic delivery of nanoparticles containing PNA and PS anti-miR-141-3p in mice after stroke. Post-treatment differentially reduced both miR-141-3p levels in brain tissue and infarct injury. We noted PNA-based anti-miR showed superior efficacy compared to PS-based anti-miR. Herein, we successfully established that nanoparticles encapsulating PNA or PS-based anti-miRs-141-3p probes could be used as a potential treatment for ischemic stroke.

## 1. Introduction

Stroke remains a leading cause of disability despite a decline in stroke-related mortality. Stroke-mediated disability imposes a substantial economic burden on individuals and society, and patients are relegated to long-term supportive care. The status quo is not adequate, and the need to develop new treatment options is imperative. MicroRNAs (miRNAs) have emerged as a target of choice due to their peculiar characteristics [1]. miRNAs are a class of short non-coding RNAs that have been identified as a potentially powerful interventional tool for many diseases, including ischemic stroke [2]. The small size of miRNA (23–25 nucleotide long) and its conserved sequence among species make it an attractive target from a drug development perspective [3].

The role of miRNA therapy in stroke has been a subject of increasing research interest since the first miRNA expression profiling study in cerebral ischemia was performed in 2008 [4]. So far, a few studies have shown synthesis and validation of stable, practical, and cost-effective modulators of these miRNAs for the treatment of stroke [5]. Anti-miRNA(anti-miR)-based therapeutics have been advanced for cancer treatment but have not been explored for stroke treatment. Among the few studies that used inhibitors to reduce stroke injury and improve chronic behavioral recovery, they have either relied on commercially available inhibitors or miRNA-based sponge. However, several challenges are needed to be overcome before their translation into the clinic. Some of these challenges include (a) Blood-brain barrier (BBB) crossing potential of therapeutic modalities, (b) Stability of anti-miRs in the circulation or brain parenchyma, and (c) Most of the prior studies relied on cerebroventricular route for administration instead of systemic administration. We have previously shown that miR-141-3p is a unique miRNA that was significantly upregulated after stroke and more so in mice isolated post-stroke in a time-dependent manner when measured up to two weeks after stroke [6]. Herein, we comprehensively study the nanoparticles delivered phosphorothioate and peptide nucleic acid-based anti-miRs-141-3p for potential stroke therapy.

Phosphorothioate (PS) is a first generation oligonucleotide in which the non-bridging oxygen of the phosphate group is substituted by sulfur, which confers its nuclease resistance features [7]. PS antisense oligonucleotides hybridize to complementary sequence by Watson Crick base-pairing rules. The PS-mRNA heteroduplex recruits RNase H enzyme that cleaves the target mRNA [8]. Recently, several PS-based antisense drugs gained FDA approval for targeting numerous diseases [9,10]. We also established that PS targeting miR-155 could be delivered by acid terminated poly (lactic-co-glycolic acid) (PLGA)-based nanoparticle formulations [11]. PLGA is a US FDA approved polymer that has been used in numerous studies for delivery of siRNA [12], plasmid DNA [13], PNAs [14], and miRNA-based mimics [15]. The electrostatic repulsion between negatively charged PS and the negatively charged acidic groups of PLGA polymers reduce efficient encapsulation of PS-based anti-miRs in the nanoparticles (NPs). We determined that PS-155, in conjunction with the nuclear localization sequence (NLS), reduces the PS-NLS encapsulant’s overall negative charge and improves the loading of the PS in the PLGA NP formulations [11]. Similarly, peptide nucleic acids are another class of nucleic acid analog that have been examined in numerous anti-miR-based applications [16,17]. Peptide nucleic acids (PNA) are synthetic DNA/RNA mimics in which the phosphodiester backbone is substituted with an achiral 2-amino-ethyl glycine unit, to provide stability and nuclease resistance [18]. PNA is neutral charged and therefore, binds to a complementary target sequence with high affinity [19].

Here, we tested both PS and PNA-based anti-miR-141-3p for stroke therapy. For delivery, we used PLGA NPs formulations. We noticed that PLGA formulations could encapsulate optimal amounts of both PS and PNA-based anti-miR-141-3p and generate NPs of superior physicochemical features; uniform spherical morphology, size, distribution and surface charge density. Further, we studied the release profile of NPs using UV-vis-based techniques and examined the significant uptake of NPs containing fluorophore tagged anti-miR. Furthermore, we established that it reduced the miR-141-3p levels after systemic delivery and reduced the infarct volume. Our work here provided a novel strategy at the interface of nucleic acid chemistry and nanotechnology to deliver a different class of anti-miRs for potential stroke therapy.

## 2. Materials and Methods

### 2.1. PNA Synthesis

Dichloromethane (DCM), N, N-dimethylformamide (DMF), pyridine, and *N*-methyl-2-pyrrolidone (NMP) were purchased from Sigma Aldrich (St. Louis, MO, USA). *N*,*N*-diisopropylethylamine (DIEA), trifluoracetic acid (TFA), diethyl ether, trifluoromethanesulfonic acid (TMFSA), thioanisole, and meta cresol were purchased from Alfa Aesar Chemicals (Ward Hill, MA, USA). Tert-butyloxycarbonyl (Boc) monomers used in PNA synthesis were purchased from ASM chemicals (Hanover, Lower Saxony Land, Germany).*O*-Benzotriazole-*N*,*N*,*N*’*N*’-tetramethyl-uronium-hexafluorophosphate (HBTU), Boc-Lys (Cl-Z)-OH, and MBHA resin was purchased from Peptide International (Louisville, KY, USA). The monomers and lysine-loaded MBHA resin was vacuum dried for two days. PNAs were synthesized in-house by Merrifield solid-phase synthesis method [20]. Lysine (K)-loaded MBHA resin was soaked in DCM overnight in the reaction vessel. The resin was deprotected using TFA- meta cresol (95:5) solution for 5 min (3×). The monomer solution was prepared by dissolving the monomer in NMP, DIEA, and HBTU solvents, and this coupling solution was added to the reaction vessel. The reaction was continued for 2 h, followed by the addition of a capping solution (NMP: pyridine: acetic anhydride). The PNA-resin bed was washed thoroughly with DCM and then proceeded to the deprotection step. All the steps were repeated, and after the last monomer of the sequence was capped, the PNA-resin was washed with DCM and deprotected with TFA for 5 min (2×). A cleavage cocktail (m-cresol, thioanisole, TMFSA, TFA, 1:1:2:6) was added to the vessel, and the cleavage was continued for 1.5 h. The PNA was collected from the vessel and precipitated using diethyl ether. The PNA was washed with diethyl ether by centrifuging the tube at 3500 rpm for 5 min at 4 °C (3×). After the last washing, diethyl ether was decanted, and the PNA was vacuum dried overnight. The PNA was purified using reverse-phase high-performance liquid chromatography (HPLC). The purity of the PNA was characterized by matrix-assisted laser desorption/ionization-time of flight (MALDI–TOF) spectroscopy (TUFTS Medical School, Boston, MA, USA). The PNA solution’s absorbance was measured at 260 nm using Nanodrop One (Thermofisher Scientific, Madison, WI, USA). Extinction coefficient of individual monomers (13,700 M^−1^cm^−1^ (A), 6600 M^−1^cm^−1^ (C), 11,700 M^−1^cm^−1^ (G), and 8600 M^−1^cm^−1^ (T)) of the sequence was used to calculate PNA concentration.

### 2.2. Thermal Melting Studies

Target miRNA 141-3p having sequence 5′ TAACACTGTCTGGTAAAGATGG 3′ was purchased from W.M. Keck Foundation Biotechnology Resource Laboratory (New Haven, CT, USA). The PS-141-3p or PNA-141-3p antimiRs were mixed with miR-141-3p target at concentration of 4 µM each. The samples were made in physiologic salt conditions and were subjected to thermal cycles from 95 °C to 25 °C and back from 25 °C to 95 °C. The absorbance of the samples was measured every 30 s at 260 nm in UV—Visible Spectrophotometer. The samples were prepared in triplicate.

### 2.3. Nanoparticle Preparation

PLGA polymers, 50:50 acid terminated polymer with 0.55 dL g^−1^ and 0.39 dL g^−1^ inherent viscosity, were purchased from Durect Corporation (Birmingham, Al, USA). The choice of PLGA polymer for encapsulating PS or PNA was made based on previous studies. Nuclear localization signal (NLS) peptide having sequence H2N-APKRKSGVSKK-OH was purchased from New England Peptide Inc. (Gardner, MA, USA). PS-based probes were purchased from Midland Certified Reagent Company (Midland, TX, USA). The PLGA NPs were formulated by double emulsion solvent evaporation technique [21]. All glassware and lab supplies required for NP formulation were sterilized before use. To prepare the organic phase 40 mg PLGA polymer was dissolved in 500 µL DCM, para-filmed securely to prevent DCM evaporation, and kept overnight. The UV-Vis absorbance of anti-miR was measured using Nanodrop One. PS-based anti-miR-141-3p (40 nanomoles) was added to 200 µL NLS peptide, and this encapsulant was added dropwise to the acid-terminated PLGA organic solution with continuous vortexing. For PNA containing NP, 40 nanomoles of PNA anti-miR was directly added dropwise to ester terminated PLGA organic solution with continuous vortexing. The resulting solution was emulsified by probe sonication (10-sec × 3 cycles) to form primary water-in-oil (w/o) emulsion. This primary emulsion was added dropwise to 1000 µL of 5% PVA w/v solution by continuous vortexing followed by probe sonication (10-sec × 3 cycles) to form a second w/o/w emulsion. This double emulsion was added dropwise to 10 mL of 0.3% PVA w/v solution and left overnight on stirring at 500 rpm to allow removal of DCM. The next day, the NPs were washed to remove excess PVA solution by centrifuging three times at 9500 rpm for 10 min at 4 °C using cold, sterile water. The NPs were re-dispersed in 5 mg/mL trehalose solution, added to sterile Eppendorf tubes, and freeze-dried (Labconco, Kansas City, MO, USA). For storage, the Eppendorf tubes were para-filmed and kept at −20 °C.

### 2.4. Scanning Electron Microscopy (SEM)

The lyophilized NPs were spread on carbon tape adhered on a stub and sputter-coated with palladium for 2 min. The images were captured under a high vacuum at 2kV accelerating voltage. The dry size of the NPs was calculated using ImageJ software (Java version 1.8, Bethesda, MD, USA).

### 2.5. Dynamic Light Scattering (DLS)

The hydrodynamic diameter, polydispersity index (PDI) and zeta potential of the NPs was evaluated using Zetasizer Nano ZS (Westborough, MA, USA). For preparing the samples for the measurements, NPs (n = 3) were dispersed in 1000 µL water followed by vortexing briefly.

### 2.6. Nucleic Acid Release Profile

The release of anti-miRs from the NPs was evaluated at 0.25, 1, 2, 4, 6, 8, 12, 24 and 48 h. Then, 300 µL phosphate buffer saline (PBS) was added to weighed NPs tubes (n = 3). The tubes were para-filmed and kept on the shaker at 300 rpm, maintained at 37 °C. At the indicated time points, the NPs were centrifuged at 15,000 rpm for 10 min at 4 °C. The supernatant was carefully withdrawn from the tubes without disturbing the NP pellet. The NP pellet was re-dispersed in 300 µL fresh PBS and kept on the shaker at 300 rpm, maintained at 37 °C until the next time point. The absorbance of the supernatant was measured at 260 nm using Nanodrop One.

### 2.7. Loading Study

A total of 200 µL DCM was added to NPs and the NPs were shaken for 3 h at 1000 rpm. Then, 200 µL of IX TE buffer was added to the NPs and further shaken for 3 h at 1000 rpm. The NPs were then centrifuged at 4 °C for 5 min at 15,000 rpm. The absorbance of the supernatant was measured at 260 nm using Nanodrop One.

### 2.8. Safety Assessment by Cell Viability Assay

About 10,000 PBMC cells (ATCC, Manassas, VA, USA) were seeded in a 96 well plate. The cells were treated with 0.02 mg, 0.04 mg, 0.08 mg, 0.1 mg and 0.2 mg PS-141 NP or PNA-141 NPs for 24 h (37° C, 5% CO_2_). The 96 well plate was kept at room temperature for 30 min. About 100 µL cell titer-Glo^®^ solution was added to each well and the plate was rocked gently for 10 min on a shaker. The luminescence was recorded using Magellan Spectrophotometer.

### 2.9. Experimental Design for In Vivo Work

A total of 26 C57BL/6 male mice (2–3-month-old) were obtained from Jackson Laboratory (Bar Harbor, ME, USA). Mice were maintained at ambient condition in the animal house with full access to food and water ad libitum. We performed animal care protocols in accordance with the Institutional Animal Care and Use Committee (IACUC) at UConn Health. Mice were pair-housed (PH) for two weeks prior to stroke surgery. Mice were examined for aggressive behavior and incompatibility between pairs of mice by measuring daily weight and eating behavior of mice. No mice were excluded due to incompatibility prior to surgery. Mice were separated immediately after stroke surgery and kept single housed. The post-surgery assigned housing conditions were maintained until animals were sacrificed.

### 2.10. Middle Cerebral Artery Occlusion (MCAO) Surgery

Transient ischemic stroke was induced by temporarily blocking the origin of middle cerebral artery as described in previous studies [6,22]. Briefly to induce transient ischemia, a ventral, midline incision was made under isoflurane anesthesia. Thereafter, the right external carotid artery was incised before inserting a 6.0 mm silicone-coated nylon filament (Doccol Corporation, Sharon, MA, USA) from the internal carotid artery bifurcation via the external carotid artery stump. Rectal temperatures were maintained at ~37 °C with the help of heating pads during the procedure. We confirm successful occlusion by laser doppler, reflecting <15% residual middle cerebral artery flow with the return of flow to 85% of baseline upon reperfusion. After an hour of occlusion, mice were perfused for predetermined time of 48 or 72 h.

### 2.11. Treatment with miR-141 Inhibitors

For in vivo efficacy, a tail vein injection of PNA-141 or PS-141 containing NPs (50 µg/kg b.w. of PNA or PS) or Scr-141 containing NPs (NPs of scrambled control) was randomly given to the mice at 4 h after stroke (ST). The successful delivery and efficacy of the treatment were determined by measuring miRNA-141-3p expression in brain tissue using qPCR. To reduce animal numbers in the study, a small tissue obtained from the perilesional cortex of 3rd brain slice was isolated and used for RNA isolation prior to TTC staining of remaining sections for infarct analysis. For biodistribution studies, NPs of anti-miR-TAMRA were injected at 24 h and 44 h after MCAO. After 48 h of MCAO onset, all the mice injected with NPs containing anti-miR-TAMRA were perfused with cold PBS and 4% paraformaldehyde and intact brains were isolated.

### 2.12. Total RNA Isolation and cDNA Synthesis

Reagents for RNA isolation, cDNA synthesis and primers of miR-141-3p were purchased from Life Technology (Life Technologies; Camarillo, CA, USA). Total RNA was extracted from perilesional ipsilateral cerebral cortex of stroked mice using the mirVana miRNA Isolation Kit (Life Technologies; Waltham, MA, USA). For miRNA expression analyses, 100 ng of cDNA was made with corresponding miRNA primer (Life Technologies; Camarillo, CA, USA) and the TaqMan Reverse miRNA Transcription Kit (Applied Biosystems; Thermofisher; Foster City, CA, USA).

### 2.13. Real-Time qPCR for miRNA Analysis

Real-time qPCR protocols were conducted using the CFX Connect™ Real-Time PCR Detection System (Bio-Rad, Hercules, CA, USA). Using cDNA samples of given miRNAs, qPCR reactions were prepared according to manufacturer’s protocols using TaqMan universal PCR master mix (Applied Biosystems; ThermoFisher; Foster City, CA, USA). miRNA 141-3- primers were used for amplification (Ambion; Life Technologies; Camarillo, CA, USA). Sno135 was used as the housekeeping gene for normalization (Ambion; Life Technologies; Camarillo, CA, USA).

### 2.14. Immunohistochemistry

Paraformaldehyde fixed and dehydrated brains (in 30% sucrose for 2–3 days) were sectioned (30 μm) on a freezing microtome and mounted on the glass side. Santa Cruz mounting media containing DAPI was used to stain Nuclei. A fluorescent microscope was used to visualize TAMRA dye and nuclei at 20× magnification in perilesional cortex of ipsilateral hemisphere.

### 2.15. Infarct Volume Analysis

For infarct analysis, 2, 3, 5 Triphenyl tetrazolium chloride (TTC) was purchased from Millipore Sigma (Burlington MA, USA). A total of three days after MCAO onset, all the mice used for infarct volume analysis were deeply anesthetized with a single injection of Avertin (250 mg/kg IP) and subjected to intracardiac perfusion with 40 mL of iced cold 1× PBS to remove blood from circulation. The intact brain was then removed and kept at −20 °C. After a brief (<5 min) period of cooling, the brain was sliced coronally at 2 mm intervals to obtain 5 total slices. Individual brain slices were then placed in 1.5% TTC solution (in 1× PBS pH 7.4) for 20 min at 37 °C in an incubator under dark conditions. Gentle stirring and flipping of the slices ensured even exposure of the surfaces to TTC staining. After 20 min, excess TTC was then drained, and brain slices were placed in a 10% formalin solution until imaged. The infarct area of each brain was measured in a blinded manner, using an image analysis software, Sigma scan (Version Pro 5). The infarct volume was calculated by Swanson’s method to correct for edema [23]. The total volumes of both contralateral and ipsilateral hemisphere in both hemispheres were measured and the percent infarct volume was calculated as % contralateral to avoid any mis-measurement secondary to edema.

## 3. Results

### 3.1. Design and Synthesis of PNA and PS-Based Anti-miR-141-3p Probes

**Figure 1:**
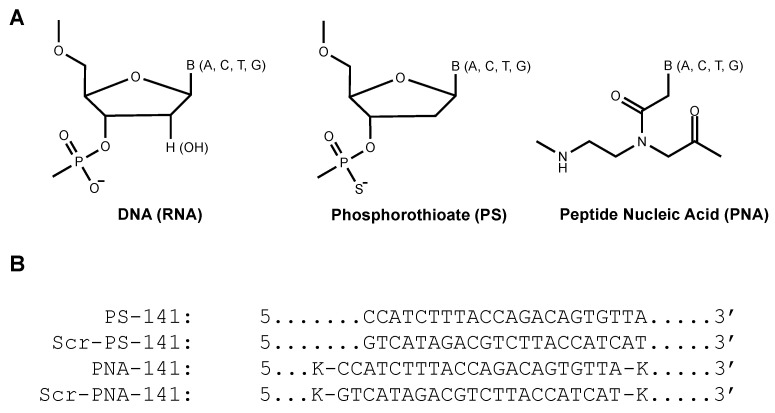
(**A**) Chemical structure of DNA and RNA, Phosphorothioate (PS) and Peptide Nucleic acid (PNA) units. DNA (RNA) and PS contain a negatively charged backbone. In comparison to DNA, in PS, non-bridging oxygen (O) of the phosphate group is replaced by a sulfur (S). PNA has a neutral backbone containing 2-aminoethyl glycine units on which the nucleobases are attached by methyl carbonyl linker. The natural nucleobases Adenine (A), Cytosine (C), Thymine (T), Guanine (G) are denoted as B. (**B**) The oligomer sequences PS-141 and PNA-141 to target miR-141-3p. Scrambled (Scr) are the controls designed for the study. Lysine residues (K) are attached at both ends of the PNAs sequences.

A prior study in C57BL/6 mice showed persistent elevated miR-141-3p levels for two weeks after stroke. miR-141-3p inhibition reduced mortality by 20% in post-stroke isolated mice. In the present research work, we designed anti-miR-PS and PNA sequences complementary to target miR-141-3p. PS-based oligomers (PS-141, Scr-PS-141, Figure 1B) were purchased commercially from Midland Certified Reagent Company. Conversely, we performed in-house synthesis of PNA oligomers (PNA-141, Scr-PNA-141, Figure 1B) using Boc-based solid-phase synthesis (Scr-PNA-141, Figure 1B) [24]. Further, quality control assessment of PNAs was performed using reverse-phase HPLC and mass spectrometry analysis.

**Figure 2:**
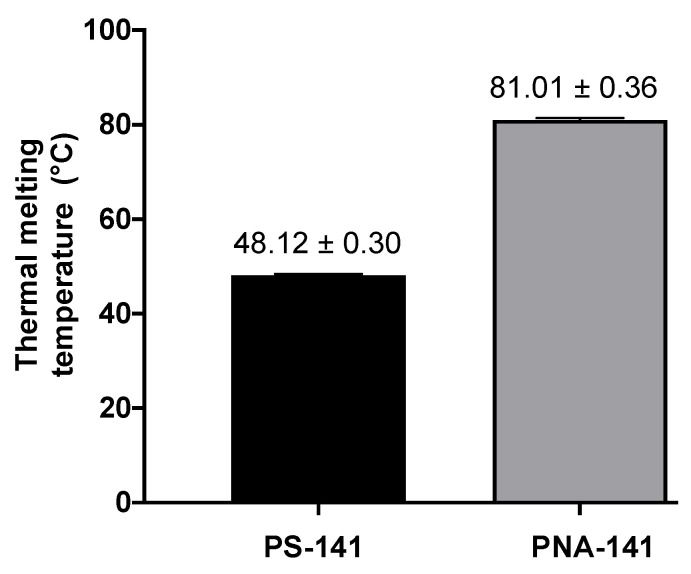
Thermal melting temperature (Tm) determination of PS-141-miR-141 duplex and PNA-141-miR-141 duplex by UV-Vis absorbance studies. The samples were made in simulated physiological buffers conditions. The concentration of anti-miRs and target miR-141-3p was 4 µM each. N = 3, data represented as mean ± standard error mean (SEM).

Scrambled PS and PNA oligomers were used as controls (Scr-PS-141, Scr-PNA-141, Figure 1B). Further, to assess the binding affinities, we performed a thermal denaturation experiment with the miR-141-3p target (Figure 2). As expected, we noted higher binding for the PNA-141-miR-141-3p duplex (81 °C) as compared to the PS-141-miR-141-3p duplex (48 °C). These findings were consistent with prior studies that documented the higher binding affinities for PNA duplex due to its neutral backbone. As expected, we did not notice any inflection point (Tm) for Scr-PS-141-miR-141-3p and Scr-PNA-141-miR-141-3p duplexes.

### 3.2. Formulating PLGA NPs and Its Characterizations

**Figure 3:**
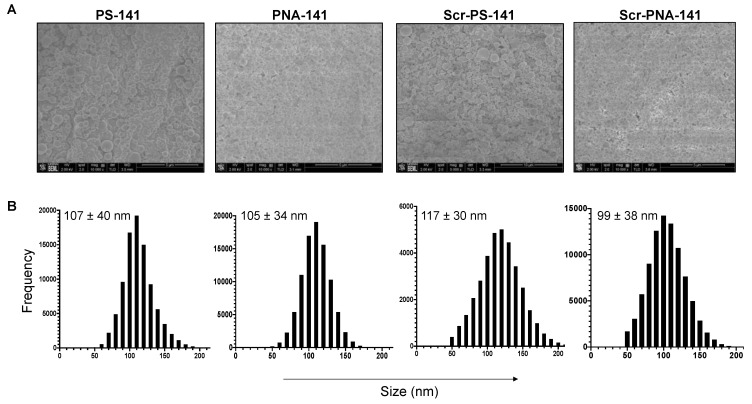
Representative SEM images showing (**A**) morphology and (**B**) size distribution curve of PLGA nanoparticles containing PS-141, PNA-141, Scr-PS-141 and Scr-PNA-141. The average particle diameter (nm) and standard deviation are given for NPs in the size distribution curve. The scale bar is 5 to 10 µm.

In prior studies, we determined that acid terminated PLGA and ester terminated PLGA NPs (containing equal ratios of poly-lactic acid and poly-glycolic acid, 50:50) can effectively deliver PS and PNA-based anti-miRs [11,25]. In this study, we sought to develop NP formulations that can encapsulate and deliver the optimum amount of PS-141 and PNA-141 and their scrambled controls. We formulated PLGA NPs containing PS-141 and PNA-141 using a double emulsion solvent evaporation technique. Blank-1 and Blank-2 NPs were generated from acid terminated and ester terminated PLGA polymer, respectively, as a control. We noticed all formulated PLGA NPs possess uniform spherical morphology as indicated by scanning electron microscopy (SEM) studies (Figure 3A). The SEM analysis showed that NPs dry size was between 100–120 nm. We performed dynamic light scattering (DLS) analyses to determine the average hydrodynamic size of formulated PLGA NPs (Table 1). The formulated NPs showed an average hydrodynamic diameter of 350 nm and an average PDI of about 0.2, indicating homogenous particle size distribution. We also observed a negative surface charge potential for all the PLGA nanoparticle formulations. The average zeta potential of the NPs was found to be −20 mV. Similarly, we did not notice any change in size, distribution, as well as zeta potential in the case of PLGA NPs containing scrambled control.

### 3.3. Nucleic Acid Release Profile

**Figure 4:**
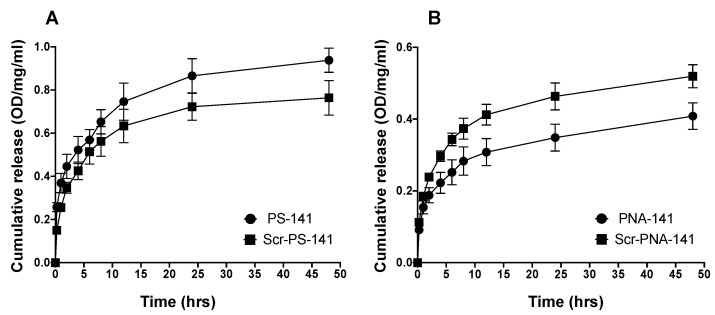
Cumulative release profile data of (**A**) PS and (**B**) PNA antimiR-141 and scramble oligomers from PLGA NPs at indicated time points (shown on the X-axis) in a graph. Data are shown as mean ± standard error mean (SEM).

**Figure 5:**
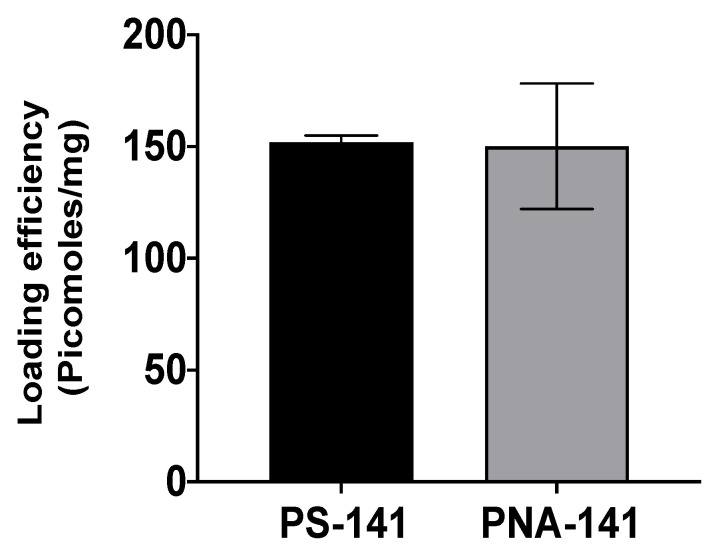
PS and PNA loading analysis. Data are shown as mean ± standard error mean (SEM).

Further, we calculated the nucleic acid release profile from PLGA NPs by re-suspending NPs in the phosphate buffer saline followed by their UV-Vis absorbance at 260 nm at indicated time points as in Figure 4. Our results indicated that the NPs showed maximum release at 24 h timepoint with a steady release of the antimiR-141-3p after 48 h. These results suggested that NPs contain a substantial amount of PS and PNA-based anti-miRs, and anti-miRs were released from NP formulation without any hindrance from the polymer. We evaluated the loading of PS-141 and PNA-141 in PLGA NPs by organic solvent extraction method. An average loading of ~170 picomoles/mg was observed for PS-141 and PNA-141 NPs (Figure 5). Overall, the loading results imply that PS and PNA antimiRs can be effectively encapsulated in PLGA NPs by double emulsion solvent evaporation technique.

### 3.4. Safety of PS and PNA NPs

**Figure 6:**
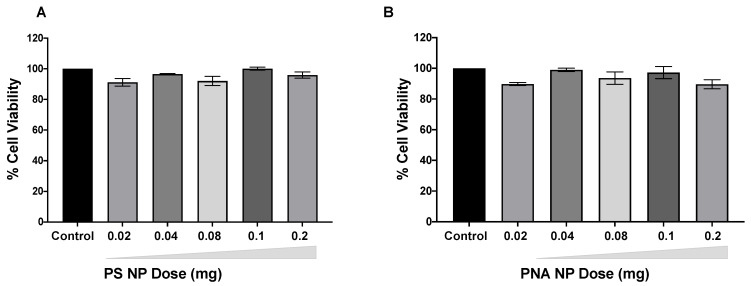
Dose dependent effect of (**A**) PS-141 NPs and (**B**) PNA-141 NPs in PBMC after 24 h. Cell viability measured by Cell Titer-Glo^®^ assay. Data are shown as mean ± standard error mean (SEM).

We next evaluated the safety of PS-141 NPs and PNA-141 NPs at doses 0.02 mg, 0.04 mg, 0.08 mg, 0.1 mg and 0.2 mg in peripheral blood mononuclear cell lines (PBMC). As expected, we did not observe any cytotoxicity for PS-141 NPs and PNA-141 NPs at all the evaluated doses (Figure 6). This suggests that the NPs are non-toxic and well tolerated even at higher dose.

### 3.5. Visualization of TAMRA Tagged PS Inhibitor in the CNS after Systemic Delivery

**Figure 7:**
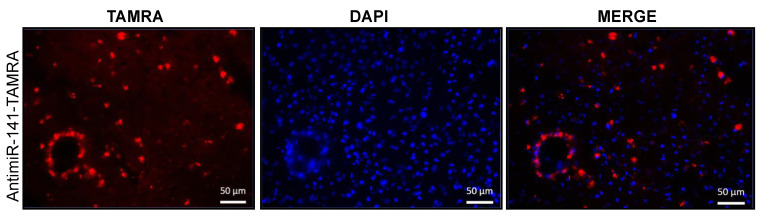
In vivo biodistribution of PS-TAMRA after tail vein injection (48 h after stroke onset) in mice (n = 4) at two days after stroke in the paraformaldehyde fixed tissue of ischemic hemisphere. Blue (nucleus; DAPI), red: antimiR-TAMRA). The scale bar is 50 µM.

To further confirm the biodistribution of anti-miRs, we formulated PLGA NPs containing Boc-5-carboxytetramethylrhodamine (TAMRA) tagged PS (PS-TAMRA). We performed two tail vein injections of PS-TAMRA in the mice after ischemic stroke. After 48 h of MCAO onset, we used paraformaldehyde-fixed tissue of the ischemic hemisphere from our stroke mouse model for confocal imaging. We noticed uniform in vivo biodistribution of TAMRA tagged PS in the brain parenchyma. Our results indicated a red fluorescent signal in the perilesional cortical area of the ischemic hemisphere (Figure 7). We also noticed that TAMRA dye-containing cell surrounding vessel-like structure in the parenchymal tissue further confirm its BBB permeability after stroke.

### 3.6. Validation of In Vivo Efficacy in Stroke Mouse Model

**Figure 8:**
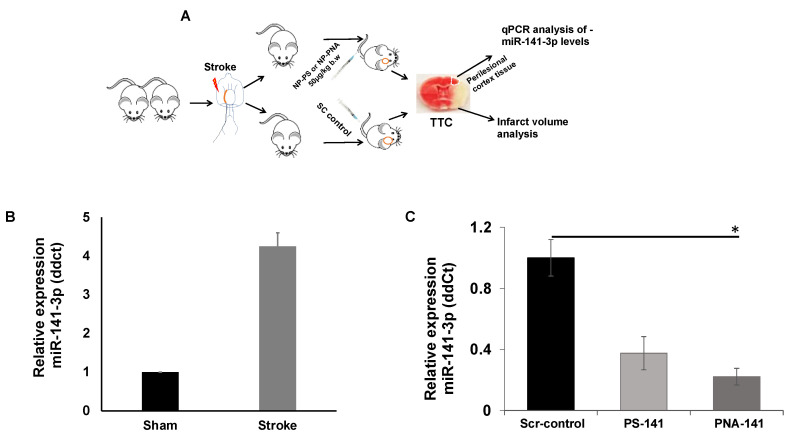
(**A**) Schematic showing in vivo work flow in mice after ischemic stroke. (**B**) Relative miR-141 gene expression analysis in RNA isolated from perilesional cortex of ischemic brain tissues and control (sham) samples (n = 3). (**C**) Relative miR-141 gene expression analysis in RNA isolated from perilesional cortex of ischemic brain tissues after systemic treatment with indicated nanoparticles on x-axis (n = 7, n = 7, and n = 8 in scrambled control, PS-NP and PNA-NP, respectively). PNA-141-3p NPs significantly (* *p* < 0.05 vs. Scr-NP) reduces miR-141-3p expression levels in brain tissue of socially isolated stroke mice. MiR-141-3p levels were optimally reduced by PS-141-3p NPs. Data are shown as mean ± standard error mean (SEM), Student T test was used for statistical analysis. * *p* < 0.05.

Further, we evaluated the efficacy of PLGA NPs containing PS-141 and PNA-141 probes in the stroke mouse model. We performed systemic delivery of PLGA NPs by tail-vein injection (Figure 8A). We also noticed that miR-141-3p upregulated in the strokes mice as compared to the control group (Figure 8B). To confirm that the PS 141-3p inhibitor crosses the BBB and de-repressed the miR-141-3p, we performed qPCR analysis of the total RNA isolated from the ipsilateral cortex of various treatment groups. We noticed that PS-NPs reduces the miR-141-3p gene expression by ~2 folds as compared to scrambled control. Conversely, the PNA-NPs-treated group shows a significant (* *p* < 0.05) ~4 folds decrease in miR-141-3p gene expression in post-stroke socially isolated mice (Figure 8C). Our results indicated the superior efficacy of PNA-NPs as compared to the PS-NPs for miR-141-3p inhibitory activity. One plausible explanation of this observation could be due to the higher binding affinity of PNA-141 with the miR-141-3p target site due to its neutral backbone. Additionally, we did not notice any mortality with the indicated dose.

### 3.7. Infarct Volume Results

TTC is a widely used dye to measure the viability of the tissue. Dead tissue remains unstained and can be easily measured to determine infarct injury after stroke. We found that after a single tail vein injection (50 µg/kg b.w.) of PNA-141 containing NPs reduced infarct volume significantly (*p* < 0.05 vs. Scr-control) compared to the scrambled control while PS-141 containing NPs showed a mild but insignificant reduction in infarct volume.

**Figure 9:**
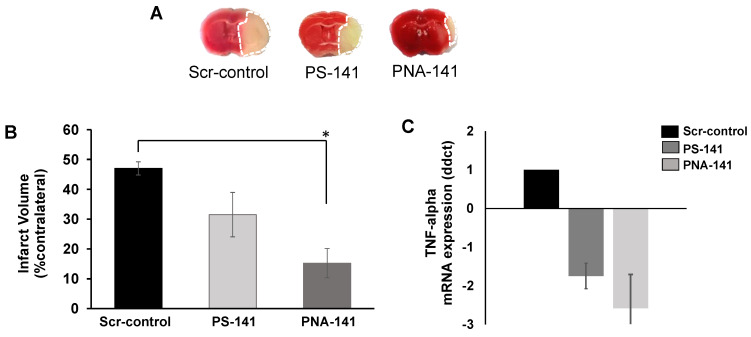
Anti-miR-141 treatment improves recovery in post-stroke socially isolated mice. (**A**) Representative coronal section of in vivo treated Scr-control (n = 7), PS-141 (n = 7), and PNA-141 (n = 8) treated brain section by TTC staining. (**B**) PNA-141 NP treatment significantly reduced infarct volume in post-stroke socially isolated mice compared to Scr-NP (* *p* < 0.05 vs. Scr-NP). PS-NP show moderate but insignificant reduction of infarct volume. (**C**) Relative gene expression of inflammatory marker TNF-alpha in the control group and NPs-treated group as indicated. Data are presented as mean ± standard error mean (SEM).

These results suggest that PNA-141 demonstrates acute neuroprotective effects by reducing miR-141-3p levels in the brain and inflammatory marker TNF-alpha (Figure 9).

## 4. Discussion

RNA-based medicines have gained enormous attention in recent years as numerous antisense-based drugs have been approved for several disorders. The majority of RNA-based drugs that acquired US FDA approval target the messenger RNA (mRNA) to control gene expression. In addition to mRNA targeting, targeting other non-coding RNAs also provides a new platform for developing therapeutic modalities to treat myriads of devastating diseases. On this front, miRNA targeting has gained immense interest due to their well-defined sequence specificity and abundant availability in the cytoplasm. Recently, miRNA-based molecular targets have been inclusively explored for cancer therapeutics and other metabolic disorders [26,27,28]. Numerous drug candidates have been investigated by miRagen and Regulus Therapeutics to target diseases like cutaneous T-cell lymphoma and Alport syndrome [29,30]. A recent study has shown that antimiR-155 has effectively targeted miRNA-155 in diffuse large B-cell lymphoma (DLBCL) patients with optimal efficacy and minimal toxicity [31]. However, miRNA-based therapeutics have not been much explored for treating other disorders such as stroke, despite their high prevalence in the US [32]. We and others have recently shown that several miRNAs are modulated by stroke and can contribute to stroke pathophysiology [4,6]. We earlier showed that stroke upregulates miR-141-3p levels in the brain and its inhibition using a commercially available miR-141-3p inhibitor improves stroke recovery in mice [6]. Given the difference in the miRNA inhibitory activity of various classes of commercially available miR-141-3p inhibitors and challenges in their delivery to the brain, it becomes imperative to identify more potent as well as BBB-permeable formulation of miR-141-3p inhibitors for the treatment of stroke. Herein, we compared the activity of two diverse classes of anti-miRs, PS and PNAs, for targeting miR-141-3p and compared their efficacy in vivo in the mouse model of stroke.

PS are well established to target mRNAs and exert RNase-based activity [33]. Similarly, PNAs have been extensively employed to target numerous miRNAs upregulated in cancers and cardiovascular disorders [34,35]. PNAs exert their mechanism of action by sterically blocking mRNA interaction with ribosomes [36]. Furthermore, PNAs are resistant to enzymatic degradation and exhibit high binding affinity with RNA sequences due to their neutral backbone [37].

However, the significant challenge associated with both PS and PNA-based anti-miR therapeutics is delivery [38]. In prior studies, PLGA nanoparticles have been used to deliver PNA-based anti-miRs for xenograft studies [39]. Recently, we optimized that acid terminated PLGA polymers can encapsulate the optimal amount of PS-based anti-miRs [11]. Furthermore, in prior studies we established that NLS could be used as a counterion to encapsulate a significant quantity of PS [11].

In general, nanoparticles take advantage of the disrupted blood vasculature of the tumor microenvironment by enhanced permeability retention (EPR) and effectively target the tumor [40]. However, for CNS-based disorders, crossing BBB possesses a major limiting factor for nanomedicine [41]. Interestingly, in stroke, the BBB gets disrupted and provides a pathophysiological advantage to the promising therapeutic modalities to cross BBB and exert their therapeutic effect [42,43].

Herein, our results indicate that PLGA NPs containing PS-141 and PNA-141 exhibit superior physico-biochemical properties, size, distribution, charge, and release profile. Further, we tested the in vivo delivery of PLGA NPs containing anti-miR in the stroked mice. Unlike a cancer-related experimental design, anti-stroke effects are difficult to assess in the cell culture assays; hence, we used systemic delivery of NPs in the well-established mouse model of ischemic stroke. To assess the BBB permeability, PS-TAMRA was injected intravenously in stroked mice and visualized on 30 µM thick brain sections by confocal microscopy. We found TAMRA containing inhibitors in the brain parenchyma, suggesting their BBB permeability. PNA-NPs significantly reduced infarct injury as measured by TTC staining. Further, after systemic delivery, PS-141 and PNA-141 nearly reduced 2-fold and 4-fold of miR-141-3p levels in the brain tissue, respectively. PNA-141 containing NPs were more effective than PS-141 containing NPs in reducing stroke injury as suggested by reduced miR-141-3p levels and infarct injury. One plausible explanation of this observation could be due to the higher binding affinity of PNA-based anti-miR 141-3p with the miR-141-3p target site due to its neutral backbone. We here showed that miR-141-3p inhibition shows its neuroprotective effects by reducing pro-inflammatory cytokine TNF-α in young adult mice after ischemic stroke. Further, enhanced neuroprotective effects and substantially reduced infarct injury by PNA-141 containing NPs might be due to improved BBB permeability and strong binding with target miR-141-3p.

## 5. Conclusions

Here we established the proof of principle study using regular PS and PNA-based anti-miRs in conjunction with PLGA technology. It will be interesting to advance this technology by using next-generation nano formulations and chemically modified nucleic acid analogs to increase its efficacy. Next-generation polymers like Poly-beta-amino-esters (PBAE), Polyethyleneimine (PEI) can generate small particles of uniform morphology and increase the CNS transfection efficiency due to their cationic features [44,45]. However, caution needs to be exercised as an increase in positive surface charge increases the cell and tissue-based toxicities [46]. Similarly, in numerous studies, it has been well documented that chemically modified gamma PNAs possess superior binding affinity than classical PNAs due to their pre-organized structure [47].

Together, our results establish that PLGA NPs containing PS-141 and PNA-141 can be used as an effective therapeutic modality for treating stroke after systemic delivery. The development of novel next generation nanoparticle-delivered chemically modified PS and PNA-based anti-miR probes may eventually lead to novel treatment regimens for stroke therapy.

## Figures and Tables

**Table 1:** Characterization of PLGA NPs containing antimiR-141 sequences. Mean diameter and polydispersity index (PDI) was calculated using dynamic light scattering (DLS), and the surface charge was calculated using zeta potential.

Nanoformulation	DLS (nm ± SEM)	PDI (± SEM)	Zeta Potential (mV ± SEM)
Blank-1	376.3 ± 09.1	0.22 ± 0.03	−21.03 ± 2.55
PS-141	349.5 ± 15.8	0.21 ± 0.02	−19.97 ± 2.71
Scr-PS-141	350.6 ± 15.8	0.20 ± 0.01	−18.83 ± 3.45
Blank-2	336.3 ± 28.3	0.21 ± 0.04	−24.17 ± 0.64
PNA-141	330.9 ± 41.4	0.18 ± 0.07	−18.03 ± 1.07
Scr-PNA-141	315.9 ± 28.1	0.18 ± 0.07	−22.03 ± 1.07
